# NEK2 affects the ferroptosis sensitivity of gastric cancer cells by regulating the expression of *HMOX1* through Keap1/Nrf2

**DOI:** 10.1007/s11010-024-04960-y

**Published:** 2024-03-19

**Authors:** Jianyong Wu, Desheng Luo, Laizhen Tou, Hongtao Xu, Chuan Jiang, Dan Wu, Haifeng Que, Jingjing Zheng

**Affiliations:** 1 Gastroenterology Department, Lishui Municipal Central Hospital, The Fifth Affiliated Hospital of Wenzhou Medical University, Lishui, Zhejiang China; 2 Gastrointestinal Surgery, Lishui Municipal Central Hospital, The Fifth Affiliated Hospital of Wenzhou Medical University, Lishui, Zhejiang China

**Keywords:** NEK2, Ferroptosis, HMOX1, Keap1/Nrf2

## Abstract

NEK2 is a serine/threonine protein kinase that is involved in regulating the progression of various tumors. Our previous studies have found that NEK2 is highly expressed in gastric cancer and suggests that patients have a worse prognosis. However, its role and mechanism in gastric cancer are only poorly studied. In this study, we established a model of ferroptosis induced by *RSL3* or *Erastin* in AGS cells in vitro, and konckdown *NEK2*, *HOMX1*, *Nrf2* by siRNA. The assay kit was used to analyzed cell viability, MDA levels, GSH and GSSG content, and FeRhoNox™-1 fluorescent probe, BODIPY™ 581/591 C11 lipid oxidation probe, CM-H2DCFDA fluorescent probe were used to detected intracellular Fe^2+^, lipid peroxidation, and ROS levels, respectively. Calcein-AM/PI staining was used to detect the ratio of live and dead cells, qRT-PCR and Western blot were used to identify the mRNA and protein levels of genes in cells, immunofluorescence staining was used to analyze the localization of Nrf2 in cells, RNA-seq was used to analyze changes in mRNA expression profile, and combined with the FerrDb database, ferroptosis-related molecules were screened to elucidate the impact of NEK2 on the sensitivity of gastric cancer cells to ferroptosis. We found that inhibition of NEK2 could enhance the sensitivity of gastric cancer cells to RSL3 and Erastin-induced ferroptosis, which was reflected in the combination of inhibition of NEK2 and ferroptosis induction compared with ferroptosis induction alone: cell viability and GSH level were further decreased, while the proportion of dead cells, Fe^2+^ level, ROS level, lipid oxidation level, MDA level, GSSG level and GSSG/GSH ratio were further increased. Mechanism studies have found that inhibiting NEK2 could promote the expression of *HMOX1*, a gene related to ferroptosis, and enhance the sensitivity of gastric cancer cells to ferroptosis by increasing HMOX1. Further mechanism studies have found that inhibiting NEK2 could promote the ubiquitination and proteasome degradation of Keap1, increase the level of Nrf2 in the nucleus, and thus promote the expression of *HMOX1*. This study confirmed that NEK2 can regulate *HMOX1* expression through Keap1/Nrf2 signal, and then affect the sensitivity of gastric cancer cells to ferroptosis, enriching the role and mechanism of NEK2 in gastric cancer.

**Supplementary Information:**

The online version contains supplementary material available at 10.1007/s11010-024-04960-y.

## Introduction

Gastric cancer is currently the fifth most newly diagnosed malignancy in the world [1], and in east Asian countries, including China, Japan and South Korea, accounts for nearly 50% of the new patients [2]. Currently, surgical resection, radiotherapy, chemotherapy, targeted therapy and immunotherapy are commonly used in clinical treatment of gastric cancer, which have a certain effect on improving the prognosis of patients. However, the current prognosis of patients with gastric cancer is still poor, with the 5-year survival rate as low as 40% [3], and the newly killed patients rank fourth among malignant tumors [1]. Therefore, new therapeutic strategies need to be developed urgently, and the revelation of their pathological mechanisms is the basis for the development of effective therapeutic strategies.

NIMA-related kinase 2 (NEK2), a member of the NEK family, is a serine/threonine protein kinase consisting of 445 amino acids, including the N-terminal catalytic kinase domain and the C-terminal regulatory domain and its, regulatory domains include Leucine zipper (LZ), centrosome and microtubule binding sites, nuclear localization signals, PP1 binding sites, etc. [4]. NEK2 can influence mitosis by regulating centrosome replication and separation, microtubule stabilization, centromere attachment, spindle assembly, etc., and is involved in a variety of physiological and pathological processes [5, 6]. In tumors, studies have found that the NEK2 expression increases in a variety of tumors, and NEK2 promotes tumor growth, metastasis, drug resistance and other processes, including breast cancer, lung cancer, colorectal cancer, etc. [7–9]. So targeting NEK2 is considered to be an ideal treatment strategy for cancers [4].

In gastric cancer, Hao Wan et al. found that NEK2 can promote aerobic glycolysis and inhibit autophagy of gastric cancer cells by activating AKT/HIF-1α signal and AKT/mTOR signal, respectively, so as to promote cell proliferation and inhibit cell apoptosis [10]. Weidong Fang et al. reported that NEK2 can promote the proliferation of gastric cancer cells by activating ERK/MAPK [11]. Yiwei Li et al. confirmed that NEK2 can promote the expression of myc through β-catenin, stabilize KDM5B protein, inhibit histone H3K4me3, and further promote the proliferation and migration of gastric cancer cells [12]. The above studies have preliminarily shown that NEK2 plays a carcinogenic role in gastric cancer, but the in-depth mechanism is unknown, which limits its clinical application value.

Ferroptosis is a Fe^2+^-dependent mode of cell death characterized by lipid peroxidation, which is different from programmed cell death such as apoptosis, necrosis and pyroptosis. It is widely involved in various pathological processes, including ischemia reperfusion, neurodegenerative diseases and tumors, etc. [13–15]. In recent years, studies have found that ferroptosis is involved in the progression of gastric cancer, tumor stemness and resistance to chemotherapy drugs, etc. For example, Guoquan Huang et al. found that BDNF-AS is highly expressed in gastric cancer tissues, suggesting a worse prognosis and inhibiting BDNF-AS can enhance the susceptibility of gastric cancer cells to Erastin and RSL3-induced ferroptosis through WDR5/FBXW7/VDAC3 [16]. Haiyang Zhang et al. found that USP7/hnRNPA1 signaling axis promotes the packaging of miR-522 into exosomes in tumor-associated fibroblasts (CAFs), and furthermore, the expression of ALOX15 was inhibited, and the ferroptosis of gastric cancer cells under the effects of cisplatin and paclitaxel decreased [17]. Targeting ferroptosis pathway is an important direction in the prevention and treatment of gastric cancer. However, whether NEK2 regulates the process of ferroptosis and thus affects gastric cancer cells is unknown.

On the basis of previous studies, this study aimed to analyze the influence of NEK2 on ferroptosis in gastric cancer, and further reveal the mechanism of NEK2 regulation of ferroptosis in gastric cancer, so as to provide data for enriching the knowledge of the mechanism of action of NEK2 in gastric cancer and to provide evidence-based medical evidence for targeting NEK2 pathway to prevent and treat gastric cancer.

## Materials and methods

### Cell lines

AGS (iCell, China, h016) were purchased from Sebikon (Shanghai) Biotechnology Co., LTD and they were used for all the dataset. AGS cells were cultured with F12K (iCell, 0007) + 5% FBS (Procell, China, 164210-500). The cells were passaged at 1:3 ratio. The mycoplasma negativity test and short tandem repeat (STR) analysis were conducted, which authenticated the cell line AGS: mycoplasma was negative for this cell line (Fig. S1A) and this cell line used in this study was real AGS cell line (Fig. S1B). Moreover, according to Cellosaurus, the cell line AGS used in this project does not contain Hela cell contamination (https://www.cellosaurus.org/CVCL_0139).

### Cell transfection

Cells in a good growth state during the logarithmic growth stage were inoculated with 2 × 10^5^ per well into a 6-well cell culture plate, cultured overnight in a 5% CO_2_ incubator at 37 °C, and then replaced with serum-free medium at 2 h before transfection. For each transfection sample, 10 μL siRNA (Genepharma) was diluted with 250 μL serum-free opti-MEM (GIBCO, #31985), gently mixed, and the mixture stood at room temperature for 5 min. At the same time, 5 μL Lipofectamine™ 2000 (Invitrogen, #11668-019) was diluted in opti-MEM and stood for 5 min at room temperature. Lipofectamine™ 2000 and siRNA diluent were mixed, the mixture stood at room temperature for 15 min, then the mixture was added into the culture well, and the cell culture plate was gently shaken back and forth to mix. The cells were cultured in a CO_2_ incubator at 37 °C. After 6 h, the mixture was sucked out and replaced with normal medium. The sequence information was as follows:

**Table:** 

Gene name (siRNA)	Sequence	
	Sense (5′–3′)	Antisense (5′–3′)
NEK2-672	GGCACACCUUAUUACAUGUTT	ACAUGUAAUAAGGUGUGCCTT
NEK2-1263	CUUCCAUCCUCAGUAAUUATT	UAAUUACUGAGGAUGGAAGTT
NEK2-417	GAUCUGGCUAGUGUAAUUATT	UAAUUACACUAGCCAGAUCTT
HMOX1-234	CCCUGUACCACAUCUAUGUTT	ACAUAGAUGUGGUACAGGGTT
HMOX1-303	CUGUCUACUUCCCAGAAGATT	UCUUCUGGGAAGUAGACAGTT
HMOX1-691	ACUGCGUUCCUGCUCAACATT	UGUUGAGCAGGAACGCAGUTT
Nrf2-1094	GCCCAUUGAUGUUUCUGAUTT	AUCAGAAACAUCAAUGGGCTT
Nrf2-809	GACAGAAGUUGACAAUUAUTT	AUAAUUGUCAACUUCUGUCTT
Nrf2-530	CCCGUUUGUAGAUGACAAUTT	AUUGUCAUCUACAAACGGGTT

### Western blot

Total protein was extracted from RIPA lysate (Beyotime, China, P0013B) containing protease inhibitor PMSF (Nanjing Wohong, China, 329-98-6), and nuclear protein was extracted with nuclear protein and cytoplasmic protein extraction kit (Beyotime, P0027). The protein concentration was determined by the Bradford method (Bio-Rad, USA, no.5000006). Subsequently, 30 μg protein was isolated by 10% SDS-PAGE and transferred to PVDF membrane (Bio-Rad, USA, no.162-0177). The PVDF membrane was incubated at 4 °C overnight with primary antibody (NEK2 antibody, abcam, ab279717; Nrf2 antibody, affnity, BF8017; keap1 antibody, abcam, ab227828; HMOX1 antibody, abcam, ab68477; GAPDH antibody, abcam, ab9485; Lamin B antibody, abcam, ab16048). The PVDF membrane was washed with TBST buffer and incubated with the secondary antibodies labeled with HRP (Dianova, Hamburg, Germany) at room temperature for 2 h, and washed with TBST and then photographed with ECL developer (Bio-Rad, USA, no.170-5060) and GelDoc imaging system (Bio-Rad). The relative protein expression levels were normalized with GAPDH as the internal reference of total protein and Lamin B as the internal reference of nuclear protein.

### qRT-PCR

Total RNA was extracted from cells using Trizol (Invitrogen life technologies, Carlsbad, CA, USA) and reverse transcribed into cDNA by reverse transcription kit. The RNA content was detected by SYBR Green method, and the reaction procedure was: 1 cycle: predenaturation at 95 °C for 10 min, 40 cycles: 95 °C denaturation for 15 s, 60 °C annealing elongation for 60 s. The results were calculated by 2^−△△CT^ method. The primers were as follows: NEK2: forward, 5′-TTGACCGGACCAATACAACA-3′, reverse, 5′-CAGGAAAACATTGGCTGGTT-3′; HMOX1: forward, 5′-ATGACACCAAGGACCAGC-3′, reverse, 5′-GTGTAAGGACCCATCGGAGA-3′; CHAC1: forward, 5′-GGTGGCTACGATACCAAGGA-3′, reverse, 5′-CCAGACGCAGCAAGTATTCA-3′; GAPDH: forward, 5′-TCAAGAAGGTGGTGAAGCAGG-3′, reverse, 5′-TCAAAGGTGGAGGAGTGGGT-3′.

### Cell viability

CCK-8 detection kit (Seven Seas Biology, 20150520) was used to detect the cell viability. The brief steps were as follows: after cell resuspension, cells were inoculated at 1 × 10^4^/well, and the uninoculated cells were used as blank control. After treatment for each group, 10 μL CCK-8 reagent was added to each well. The absorbance at 450 nm of each well was determined by Multiskan MK3 enzyme spectrometer (MD, Spectramac M3), and statistical analysis was performed.

### Fe^2+^ level detection

After the cell culture medium was removed, the cells were rinsed with PBS and fixed with 4% paraformaldehyde at 4 °C for 15 min, followed treated by 5% BSA for 30 min. Then the cells were treated with FeRhoNox™-1 fluorescent probe (Goryo, Japan, GC901) with final concentration of 5 μM at 37 °C for 30 min. After washing with PBS, the cells were stained with DAPI, and the Fe^2+^ level was detected by flow cytometry (Beckmancoulter, USA, USA).

### ROS

The cells were centrifuged at 1500 rpm for 5 min, then rinsed with PBS, then treated with CM-H2DCFDA fluorescent probe diluted according to the instructions (Solarbio, China, D6470) at 37 °C for 20 min, then washed with serum-free medium, and then suspended with PBS. Flow cytometry (Beckmancoulter, USA, cytoFLEX) was used to detect ROS levels.

### Lipid oxidation levels

Cells were collected after digestion with pancreatic enzymes, rinsed with PBS, then treated with BODIPY™ 581/591C11 lipid oxidation probe (Invitrogen, USA, D3861), which was diluted according to the instructions, at 37 °C for 20 min, then the cells were washed with serum-free medium, and suspended with PBS. Flow cytometry (Beckmancoulter, USA, cytoFLEX) was used to detect the level of cellular lipid oxidation.

### MDA detection

MDA detection kit (Beyotime, China, S0131S) was used to detect MDA levels. The brief steps were as follows: 0.1 mL homogenate was added into the centrifuge tube as the blank control, 0.1 mL standard substance of different concentrations (1, 2, 5, 10, 20, 50 µM) was added to make the standard curve, and 0.1 mL sample was added for determination. Then 0.2 mL MDA detection liquid was added to each centrifuge tube, mixed well, heated in a boiling water bath for 15 min, cooled to room temperature, centrifuged at 1000×*g* for 10 min, and 200 μL supernatant was added to the 96-well plate. The absorbance of 532 nm was measured by the microplate reader. The MDA concentration was calculated according to the standard curve. The MDA content in the initial sample was expressed by the protein content per unit weight in µmol/mg.

### GSH and GSSG levels

GSH and GSSG levels were measured using the GSH and GSSG test kit (Beyotime, China, S0053). The brief steps were as follows: the working solution and sample were prepared, and then the sample and standard were added and mixed well. After adding 150 μL total glutathione detection solution, the sample solution was incubated at room temperature for 5 min. Then 50 μL 0.5 mg/mL NADPH solution was added and mixed well. After 25 min, the A412nm of the sample was determined by the microplate reader and the total glutathione was calculated. The GSH in the sample was removed with appropriate reagent, and then the GSSG content was determined by the reaction principle mentioned above. The GSSG content was subtracted from the total glutathione (GSSG + GSH) to obtain the GSH content result.

### Detection of living/dead cells by Calcein-AM/PI

The operating instructions of Calcein-AM/PI living/dead cell double staining kit (Yi Sheng Bio, 40747ES7) was followed. The brief steps were as follows: 10× Assay Buffer was diluted with deionized water (dH_2_O) to obtain 1× Assay Buffer. 5 μL Calcein-AM solution and 15 μL PI solution were added to 5 mL 1× Assay Buffer. After fully mixing, the Calcein-AM working fluid and PI working fluid were prepared. Cells were then fully rinsed with 1× Assay Buffer and then re-suspended to an density of 5 × 10^5^ cells/mL. 100 μL staining solution and 200 μL cell suspension were mixed and incubated at 37 °C for 15 min. Detection by flow cytometry (BECKMAN, CytoFLEX) was finally performed.

### RNA-seq

Cells were collected and mRNA expression profiles were detected by RNA-seq. In the sequencing results, fold change (FC) ≥ 2 or FC ≤ 0.5 (that is, the absolute value of log_2_FC ≥ 1) was used as the change threshold, and *q* value < 0.05 (*q* value was the correction value of *p* value) was used as the standard for screening differential genes (|log_2_FC| ≥ 1&*q* < 0.05). In the group for comparison, the results of differential expression gene analysis, differential expression gene GO enrichment analysis, differential expression gene KEGG pathway enrichment analysis were obtained.

### Immunofluorescence staining

The cells were inoculated in petri dishes covered with coverslip, treated according to the grouping, and the cells were collected, rinsed and fixed with 4% paraformaldehyde for 15 min, permeated with 0.5% Triton X-100 for 20 min, and sealed with goat serum for 30 min. Then the diluted primary antibody (Nrf2, proteintech, 16396-1-AP) was added and the cells were incubated at 4 °C overnight. After the washing with PBST, Cy3-labeled Sheep Anti-Rabbit Fluorescent Secondary Antibody IgG (Wuhan Bode Bioengineering Co., LTD., BA1032) was added and the cells were incubated at room temperature for 2 h in darkness. After the washing with PBST, the cells were stained with DAPI (Beyotime, C1002), and sealed with anti-fluorescent quencher. The images were taken under fluorescence microscope (Olympus BX53 biological microscope).

### Statistical analysis

Three biological replicates were available for all data. Data analysis was performed using GraphPad Prism 9.0 statistical software. All data were expressed as mean ± standard deviation, and the comparison between different groups was performed using LSD method (least significance method) in One-way ANOVA. *P* < 0.05 was considered statistically significant.

## Results

### Inhibition of NEK2 enhanced the ferroptosis sensitivity of gastric cancer cells

Preliminary studies have shown that NEK2 plays a oncogene role in gastric cancer [10–12], and our previous studies have found that NEK2 was highly expressed in gastric cancer tissues and suggested that patients have a worse prognosis [18]. Ferroptosis is a Fe^2+^-dependent programmed cell death characterized by lipid peroxidation accumulation. To determine whether inhibition of NEK2 affects the ferroptosis sensitivity of gastric cancer cells, we knockdown NEK2 expression and induced ferroptosis in AGS cell line. AGS cell mycoplasma detection and STR identification can be found in supplementary data (Fig. S1).

**Fig. 1:**
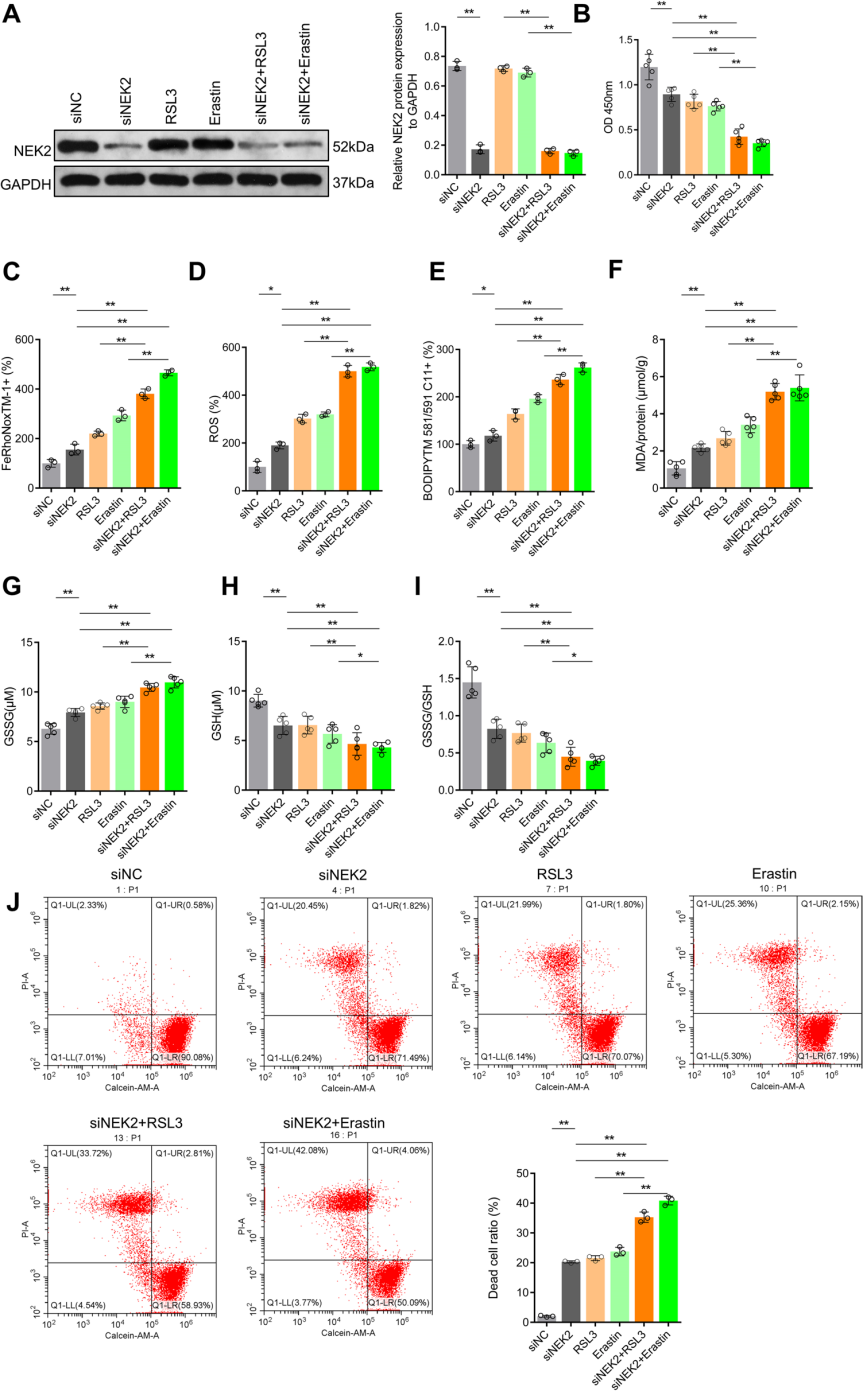
Inhibition of *NEK2* enhanced the ferroptosis sensitivity of gastric cancer cells. **A** Western blot and quantitative analysis of NEK2 levels. The cells were treated with 10 μM Erastin or 32 μM RSL for 12 h; **B** CCK-8 was used to detect and analyze cell viability. **C** FeRhoNox™-1 fluorescent probe determined the level of Fe^2+^; **D** CM-H2DCFDA fluorescent probe was used to detect ROS levels in cells; **E** BODIPY™ 581/591 C11 lipid oxidation probe was used to analyze the level of intracellular lipid oxidation; **F** The MDA level of oxidative product was detected by the kit. The levels of GSH (**G**) and GSSG (**H**) in cells were analyzed and their ratios (**I**) were calculated; **J** The proportion of living and dead cells was measured by Calcein-AM/PI staining. *Represents *p* < 0.05, **represents *p* < 0.01

Firstly, it was found that the ferroptosis activator RSL3 and the ferroptosis inducer Erastin did not affect the level of NEK2 (Fig. 1A). Cell viability experiments showed that inhibition of NEK2 or treatment with RSL3 and Erastin significantly decreased cell viability. After AGS was treated with RSL3 or Erastin on the basis of konckdown NEK2, cell viability decreased significantly, more than that treated with RSL3 or Erastin alone (Fig. 1B). According to the analysis of Fe^2+^ levels, it was found that inhibiting NEK2 or treating AGS with RSL3 and Erastin increase Fe^2+^ levels, while treating AGS with RSL3 or Erastin on the basis of the konckdown of NEK2 had a more significant increase in Fe^2+^ levels (Fig. 1C). Further observation of the changes in the oxidation level showed that the ROS, lipid peroxidation and MDA levels were also increased when NEK2 was inhibited alone or treating AGS with the RSL3 and Erastin. When the cells were treated with RSL3 or Erastin on the basis of NEK2 knockdown, ROS, lipid peroxidation and MDA levels were further increased (Fig. 1D–F). The levels and ratios of reduced glutathione (GSH) and oxidized glutathione (GSSG) in the cells were also analyzed, and it was found that GSH levels decreased while GSSG and GSSG/GSH ratios increased when NEK2 was inhibited alone or treating AGS with the RSL3 and Erastin. After the cells were treated with RSL3 and Erastin on the basis of NEK2 knockdown, GSH levels were further reduced, while GSSG and GSSG/GSH ratio were further increased (Fig. 1G–I). Finally, the conditions of living and dead cells in the cells were detected, and it was found that the level of living cells decreased while the proportion of dead cells increased when NEK2 was inhibited alone or treating AGS with the RSL3 and Erastin. The level of living cells further decreased and the proportion of dead cells increased further when NEK2 was inhibited together with the RSL3 and Erastin treatment (Fig. 1J). These results suggested that inhibiting NEK2 could activate ferroptosis activity of gastric cancer cells and enhance the ferroptosis sensitivity of gastric cancer cells.

### Inhibition of NEK2 promoted the expression of *HMOX1*

**Fig. 2:**
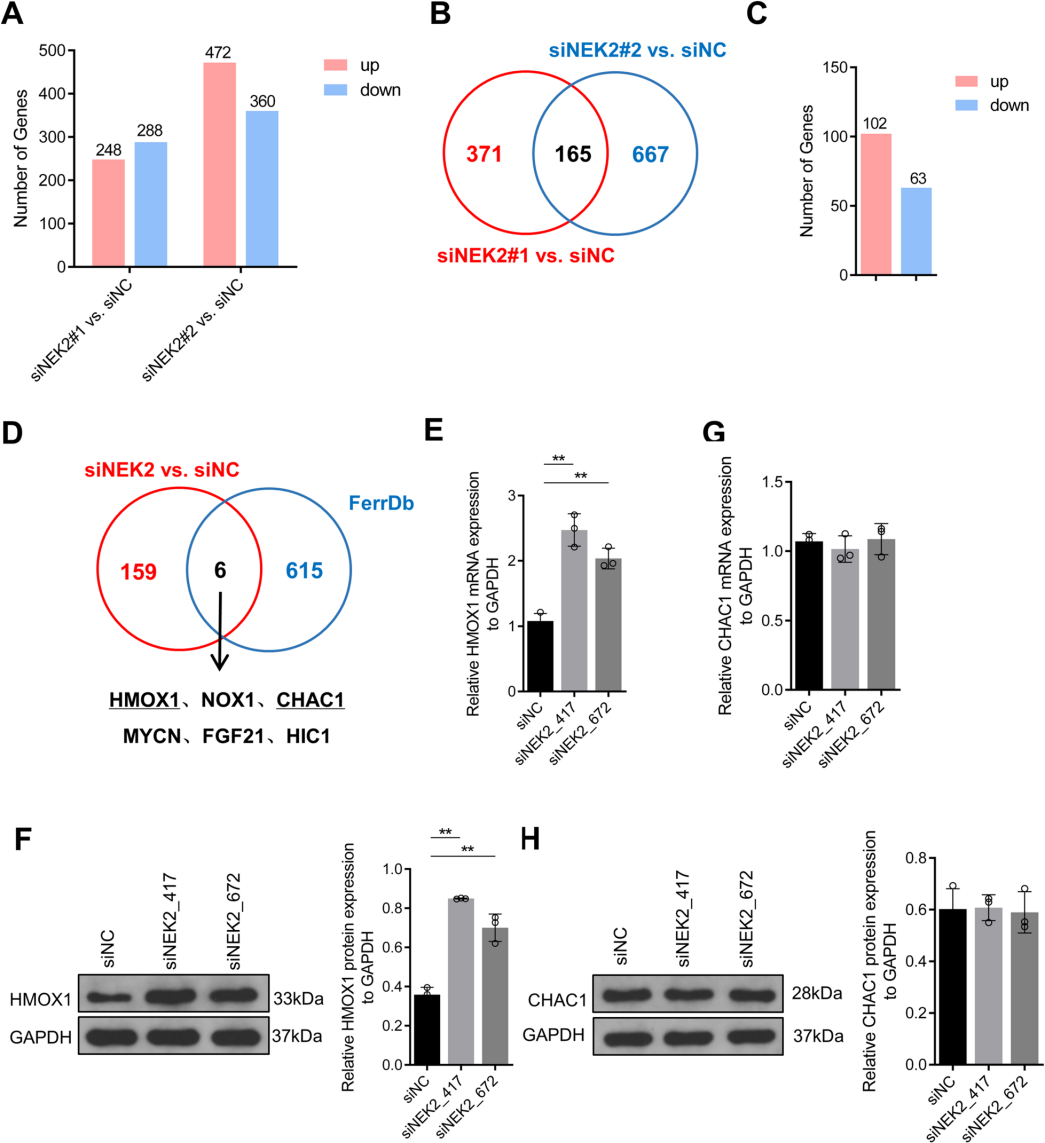
Inhibition of *NEK2* increased the expression of ferroptosis-related gene HMOX1 in gastric cancer. **A** Statistical analysis for the differentially expressed genes in the cells with inhibition of NEK2 based on RNA-seq; **B** Venn diagram showed the number of genes with consistent changes in different siRNA to knockdown NEK2; **C** The number of up-regulated and down-regulated genes with consistent changes; **D** The Venn diagram showed the number of overlapping genes between differentially expressed genes and ferroptosis-related genes in FerrDb; **E** qRT-PCR was used to detect HMOX1 mRNA levels. **F** Western blot and quantitative analysis of HMOX1 protein level in cells; **G** The mRNA level of *CHAC1* was detected by qRT-PCR. **H** Western blot and quantitative analysis of CHAC1 protein level. *Represents *p* < 0.05, **represents *p* < 0.01

In order to reveal the mechanism of NEK2 knockdown enhanced the ferroptosis sensitivity of gastric cancer cells, we analyzed the differentially expressed genes in samples after inhibiting NEK2 based on RNA-seq, and screened differentially expressed genes by setting the |log_2_FC| ≥ 1&*q* value < 0.05. In Fig. 2A, siNEK2#1 and siNEK2#2 represent different clones resulting from the same siRNA transfection. The results showed that there were 536 differentially expressed genes in siNEK2#1 group, of which 248 were up-regulated and 188 were down-regulated. There were 832 differentially expressed genes in siNEK2#2 group, among which 472 were up-regulated and 360 were down-regulated (Fig. 2A). The intersection and same changes genes were screened, a total of 165 genes, of which 102 were up-regulated and 63 were down-regulated (Fig. 2B, C). To analyze whether NEK2 regulated ferroptosis pathway gene expression, we intersected these differentially expressed genes with those genes associated with ferroptosis from the FerrDb website (http://www.zhounan.org/ferrdb/current/). Six genes were screened out and they were *HMOX1*, *NOX1*, *CHAC1*, *MYCN*, *FGF21* and *HIC1*, respectively (Fig. 2D). Considering that the signal of molecules with FPKM value < 10 was weak, it is not recommended to verify them. Therefore, *HMOX1* and *CHAC1* molecules with FPKM value ≥ 10 are selected for verification. The results showed that inhibiting NEK2 expression increased *HMOX1* mRNA and protein levels in gastric cancer cells (Fig. 2E, F), consistent with the results of RNA-seq, while CHAC1 levels did not change (Fig. 2G, H).

### Inhibition of NEK2 enhanced the ferroptosis sensitivity of gastric cancer cells by increasing HMOX1

**Fig. 3:**
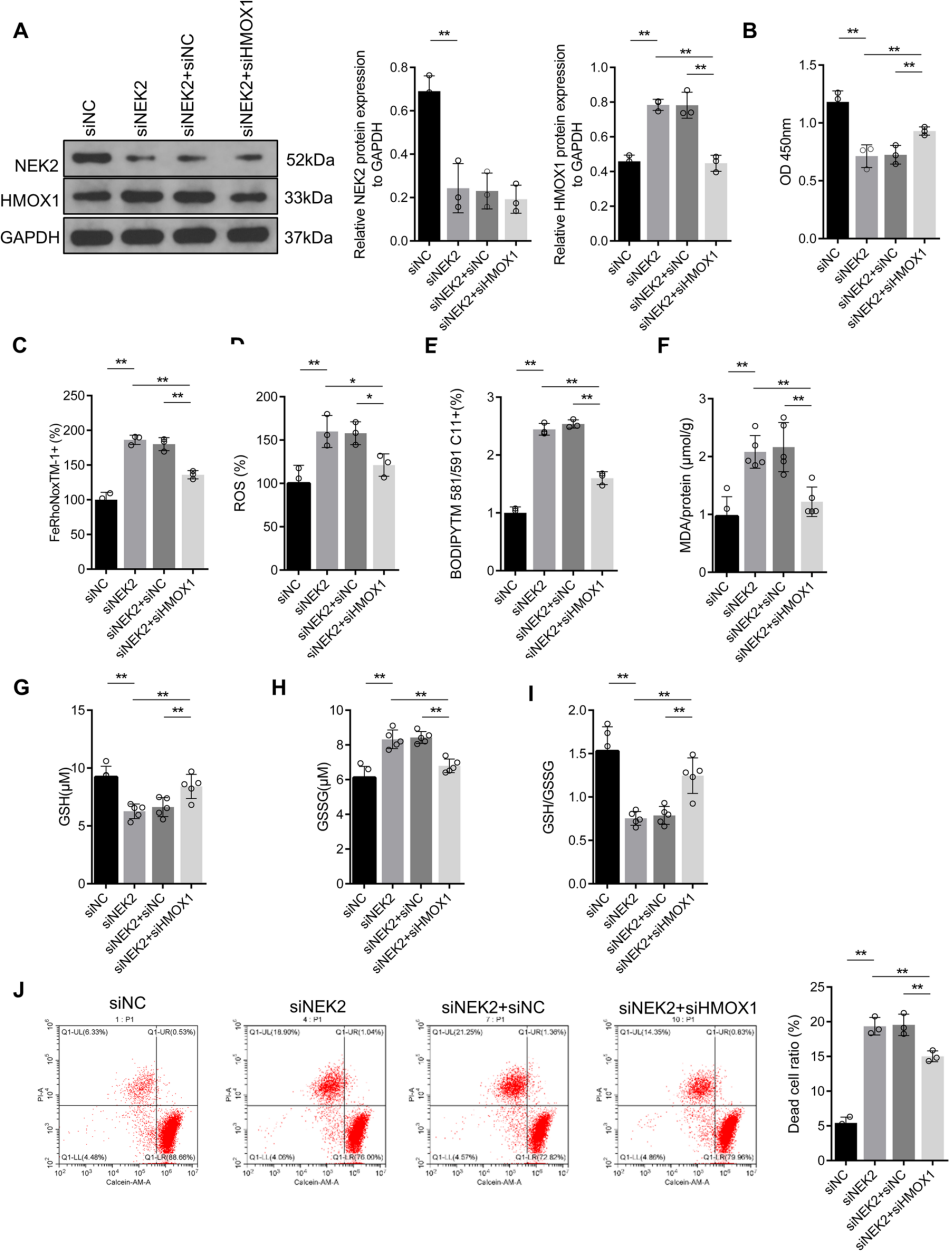
Inhibition of *NEK2* enhanced the ferroptosis sensitivity of gastric cancer cells by increasing HMOX1. **A** Western blot and quantitative analysis of NEK2 and HMOX1 levels; **B** CCK-8 analysis of cell viability; **C** FeRhoNox™-1 fluorescence probe determined the change of Fe^2+^ level. **D** The ROS levels in the cells were analyzed by CM-H2DCFDA; **E** the BODIPY™ 581/591 C11 lipid oxidation probe was used to detect the level of intracellular lipid oxidation. **F** The MDA level was detected by the kit. The levels of GSH (**G**) and GSSG (**H**) in cells were analyzed and their ratios (**I**) were calculated. **J** The proportion of living and dead cells was measured by Calcein-AM/PI staining. **Represents *p* < 0.01

As a rate-limiting enzyme in the catabolism of heme, HMOX1 can decompose heme into CO, Fe^2+^ and biliverdin, which plays a dual role in ferroptosis. On one side, it can antagonize ferroptosis by inhibiting oxidation, and on the other side, excessive production of Fe^2+^ and ROS can promote ferroptosis [19]. In order to determine whether NEK2 regulates the ferroptosis sensitivity of gastric cancer cells through HMOX1, we inhibited the expression of *HMOX1* on the basis of *NEK2* knockdown in vitro, and the results showed that inhibiting *HMOX1* on the basis of *NEK2* knockdown, the cell viability increased compared with *NEK2* knockdown alone (Fig. 3A, B), and the level of Fe^2+^ showed the same change (Fig. 3C). The analysis of oxidation levels also showed that inhibiting *HMOX1* expression on the basis of *NEK2* knockdown significantly reduced ROS levels, lipid peroxidation and MDA level compared with interfering with *NEK2* alone (Fig. 3D–F). The detection of GSH and GSSG levels showed that GSH levels significantly recovered, and GSSG and GSSG/GSH ratio decreased in the group inhibiting *HMOX1* and *NEK2* together, compared with the *NEK2* knockdown alone (Fig. 3G–I). Finally, the proportion of living and dead cells in the cells was studied, and it was shown that the proportion of living cells increased and the proportion of dead cells decreased in the group inhibiting *NEK2* and *HMOX1* together, compared with those in the cells of inhibiting *NEK2* alone (Fig. 3J). These results confirmed that inhibition of *NEK2* could enhance the ferroptosis sensitivity of gastric cancer cells by increasing *HMOX1* expression.

### Inhibition of NEK2 enhanced HMOX1 expression in gastric cancer cells through Keap1/Nrf2

**Fig. 4:**
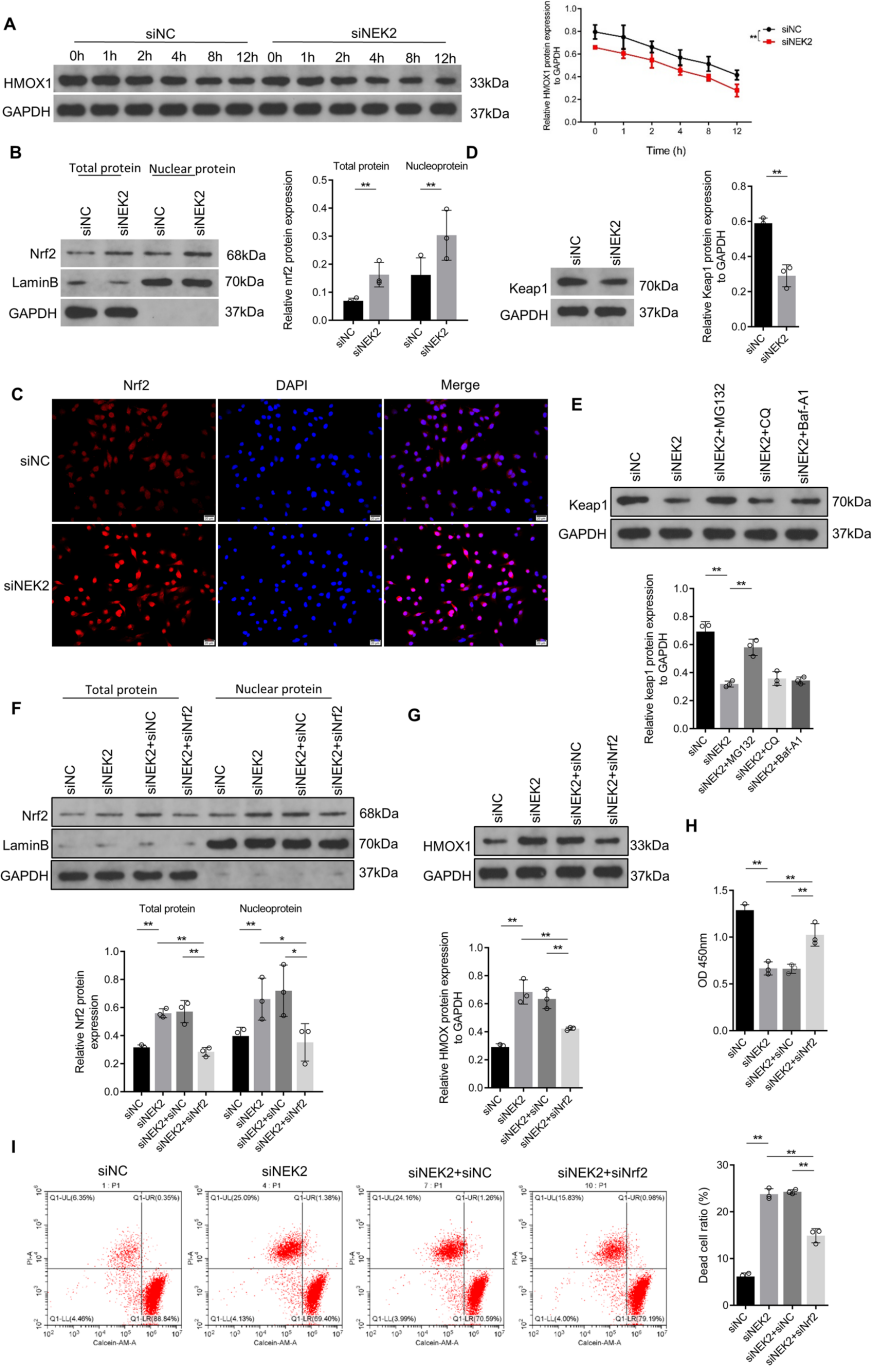
Inhibition of *NEK2* increased *HMOX1* expression in gastric cancer cells through Keap1/Nrf2. **A** After 10 μg/mL CHX treatment for 0 h, 1 h, 2 h, 4 h, 8 h and 12 h, Western blot detection and quantitative analysis of HMOX1 level were performed; **B** after extraction of total protein and nuclear protein, Western blot and quantitative analysis of Nrf2 were performed. Lamin B was used as the internal reference of nuclear protein and GAPDH was used as the internal reference of total protein; **C** the staining intensity and localization of Nrf2 were analyzed by immunofluorescence staining; **D** Western blot analysis and quantitative analysis of Keap1 level; **E** Western blot assay and quantitative analysis of Keap1 levels. The cells were treated with 10 μM MG132, 25 μM CQ, 100 nM Baf-A1 for 6 h; **F** Western blot and quantitative analysis of Nrf2 in total protein and nuclear protein; **G** Western blot and quantitative analysis of HMOX1 level; **H** CCK-8 was used to detect cell viability; **I** Calcein-AM/PI staining was used to detect the proportion of living and dead cells. *Represents *p* < 0.05, **represents *p* < 0.01

Next, we intended to reveal the mechanism of elevation of HMOX1 by *NEK2* knockdown. Under various physiological and pathological conditions have shown that HMOX1 is regulated by Keap1/Nrf2. Under oxidative stress, keap1 degradation increases, promotes Nrf2 entry into the nucleus and promotion of *HMOX1* expression. In order to determine whether NEK2 in gastric cancer cells regulates *HMOX1* expression through Keap1/Nrf2 signaling and then affect the ferroptosis sensitivity, firstly, we analyzed the effect of *NEK2* on the degradation of HMOX1 protein, and the results showed that *NEK2* knockdown did not affect the degradation level of HMOX1 protein (Fig. 4A), suggesting that NEK2 might regulate the synthesis process of HMOX1. Further detection of Nrf2 level showed that after *NEK2* konckdown, Nrf2 levels in total protein and nuclear protein of gastric cancer cells increased (Fig. 4B); immunofluorescence staining also showed an increase in overall fluorescence intensity and fluorescence intensity in nucleus (Fig. 4C); quantitative analysis of Keap1 level showed that after *NEK2* inhibition, Nrf2 levels increased and Keap1 levels were significantly decreased (Fig. 4D), suggesting that NEK2 might regulate *HMOX1* expression through Keap1/Nrf2, and the mechanism was related to reducing Keap1 expression, increasing Nrf2 level in nucleus, and thus promoting *HMOX1* expression.

Proteasome inhibitor MG132 and autophagy inhibitors Chloroquine (CQ) and Baf-A1 were used to analyze the mechanism of *NEK2* regulation Keap1 level, and it was found that Keap1 level recovered significantly after MG132 was added. However, adding CQ or Baf-A1 did not affect Keap1 levels (Fig. 4E), indicating that the mechanism of inhibiting NEK2 to reduce Keap1 levels was related to the activation of the ubiquitination-proteasome pathway.

Finally, we analyzed the effects of inhibiting *NEK2* on the HOMX1 level, cell viability, cell death level through Nrf2. The results showed that HMOX1 levels decreased after inhibiting NEK2 and Nrf2 together compared with *NEK2* knockdown alone (Fig. 4F, G). Cell viability analysis also showed that compared with inhibition of *NEK2* alone, combined intervention of and NEK2 and Nrf2 significantly restored cell viability (Fig. 4H). Flow cytometry after staining showed that compared with inhibition of *NEK2* alone, combined intervention of NEK2 and Nrf2 increased the proportion of living cells and decreased the proportion of dead cells (Fig. 4I). Combined with the previous results, it was shown that inhibiting NEK2 could promote the expression of HMOX1 through Keap1/Nrf2, enhance the ferroptosis sensitivity of gastric cancer cells, and further affect the cell biological function.

## Discussion

Gastric cancer is one of the most deadly malignancies, especially in east Asia. Despite great efforts to control the causes of the disease, improve lifestyle, and advance screening, the incidence of gastric cancer is still high and the prognosis is poor [20]. Revealing the pathological mechanism of gastric cancer is very important for developing new therapeutic strategies for gastric cancer. In this study, it was found that inhibiting *NEK2* could increase the ferroptosis sensitivity of gastric cancer cells. On the mechanism, it was found that NEK2 could regulate the process of ferroptosis by promoting keap1 ubiquitination and proteasome degradation, enhancing Nrf2 level and into the cell nucleus, promoting *HMOX1* expression.

As a serine/threonine protein kinase, NEK2 is involved in the regulation of microtubule stability, centrosome replication and separation, and chromatin agglutination [21]. In recent years, studies focus on the roles of protooncogenes of *NEK2*. It was reported that NEK2 activates YAP signals by interacting with striating-interacting phosphatase and kinase (STRIPAK) complex, promotes the expression of target genes *CTGF*, *CYR61* and *GLI2*, thereby promoting the proliferation of cervical cancer cells [22]. In glioblastoma, NEK2 increases the stability and activity of NIK through phosphorylation, thereby activating non-classical NF-κB signaling and promoting tumor progression [23]. In Diffuse large B-cell lymphoma (DLBCL), NEK2 can enhance the stability of PKM2 by binding and phosphorylating, promoting cell proliferation and glycolysis [24]. In pancreatic cancer, NEK2 can enhance its stability through phosphorylation of PD-L1, and inhibition of *NEK2* can increase lymphocyte infiltration in tumor tissue, enhance anti-tumor immune response and sensitivity to immunotherapy [25]. In cervical cancer, NEK2 activates β-catenin signaling by promoting wnt1 expression, enhancing cell resistance to radiotherapy [26]. These studies fully illustrate the diversity and richness of the roles and mechanisms of *NEK2* in tumor progression.

However, an experiment based on 19 cell lines, including gastric cancer, leukemia, colorectal cancer, prostate cancer, breast cancer, and liver cancer, found that gastric cancer cell lines were more sensitive to the NEK2 inhibitor MBM-5 [27], suggesting that NEK2 plays a more critical role in gastric cancer progression. However, currently except that NEK2 is found able to regulate gastric cancer progression through AKT/HIF-1α, AKT/mTOR, ERK/MAPK and β-catenin/myc/KDM5B signaling, only Gong Kunmei et al. found that circPDSS1/miR-86-5p/NEK2 signal could promote the progression of gastric cancer [28]. Therefore, there are few studies on the mechanism of action of NEK2 in gastric cancer, which is one of the key factors limiting the development of therapeutic strategies targeting NEK2. Our previous studies confirmed that *NEK2* is highly expressed in gastric cancer and is associated with patient prognosis, and analysis found that high expression of *NEK2* indicates reduced immune cell infiltration in tissues, lower anti-tumor immune activity, and more sensitive to drugs targeting cell cycle and DNA replication pathways [18]. This study found that inhibiting *NEK2* can increase the ferroptosis sensitivity of gastric cancer cells, and enrich the mechanism of NEK2 in gastric cancer. In recent years, the relationship between ferroptosis and cancer has become the focus of research, and targeting ferroptosis is an important direction of tumor prevention and treatment [29]. In gastric cancer, Shunhong Mao et al. found that miR-489-3p can mediate levobupivacaine-induced ferroptosis of gastric cancer cells by targeting SLC7A11 [30]. Perilipin-2 may be involved in gastric cancer progression by regulating abnormal lipid metabolism and ferroptosis [31]; polyunsaturated fatty acid biosynthesis pathway is associated with ferroptosis sensitivity of gastric carcinoma cells [32]. These studies confirmed a strong link between gastric cancer and ferroptosis. This study established the association between *NEK2* and ferroptosis for the first time, further enriching the mechanism of ferroptosis in gastric cancer.

The mechanism of inhibiting *NEK2* to enhance the sensitivity of gastric cancer cells to ferroptosis is related to promoting the expression of *HMOX1*. Currently, it has been found that HMOX1 can catalyze the production of CO, Fe^2+^ and biliverdin from heme, and can inhibit and promote ferroptosis. Activation of HMOX1 in physiological state can play a role in clearing ROS and protecting cells, while over-activation of HMOX1 can increase iron accumulation, and free Fe^2+^ has a high oxidation type and it is prone to Fenton reaction with H_2_O_2_ to produce more toxic oxygen species, such as hydroxyl radical and hydrogen peroxide, which leads to damage of DNA, protein and membrane lipids, promotes lipid peroxidation and damages the cell membrane and induce ferroptosis [19, 33, 34]. In this study, we found that inhibiting *NEK2* increased HMOX1 and played a role in promoting ferroptosis. The promotion of ferroptosis by HMOX1 has been confirmed in various pathological processes, such as the high expression of *HMOX1* in diabetic atherosclerosis. Inhibition of *HMOX1* can reduce the levels of Fe^2+^ and ROS in cells, increase the levels of SLC7A11 and GPX4, and delay lipid peroxidation [35]. EF24, 3,5-bis (2-fluorobenzylidine)-4-pyperidone, is a synthetic analogue of curcumin and Zoledronic acid to promote ferroptosis of osteosarcoma cells by inducing HMOX1 [36, 37]. Tagitinin C promotes ferroptosis in colorectal cancer cells by inducing ER stress to activate Nrf2/HMOX1 signaling [38]. DpdtbA (2,2′-di-pyridineketone hydrazone dithiocarbamate butyric acid ester) can promote ferrroptosis of gastric cancer cells through with Keap1/Nrf2/HMOX [39]. In this study, we found that inhibiting *NEK2* could increase the levels of Fe^2+^, ROS and lipid peroxidation, and these phenomena were recovered after *HMOX1* knockdown, confirming that inhibiting *NEK2* and then increasing HMOX1 promote the process of ferroptosis in gastric cancer, which is related to excessive Fe^2+^ accumulation.

The expression of *HMOX1* can be regulated by Keap1/Nrf2, and keap1 level in cells is highly susceptible to oxidative stress. Under oxidative stress, keap1 can be degraded through ubiquitination-proteasome pathway, which increases the stability and nucleation level of Nrf2 and induces downstream gene expression. This mechanism is particularly obvious during ferroptosis. For example, 4,4′-dimethoxychalcone was found able to promoting ferroptosis in cancer cells by promoting the keap1 ubiquititation-proteasome degradation pathway, thereby activating the Nrf2/HMOX1 signaling axis [40]. In this study, when we analyzed the mechanism of NEK2 regulating *HMOX1* expression, we found that NEK2 could affect the ubiquitination-proteasome degradation pathway of keap1. Although previous studies have found that NEK2 can affect the autophagy level in gastric cancer cells [10, 41], in this study, we found that NEK2 did not regulate keap1 through the autophagy pathway. Moreover, although previous studies have shown that oxidative stress can promote the ubiquititation-proteasome degradation of Keap1, and inhibition of *NEK2* in this study could increase ROS levels in cells, many studies have reported that NEK2 can regulate the ubiquititation-proteasome degradation pathway based on molecular interaction mechanisms. For example, in pancreatic cancer, NEK2 can inhibit ubiquitination-proteasome degradation by binding to PD-L1 and phosphorylating its T194/T210 sites [26]. In hepatocellular carcinoma, NEK2 inhibits ubiquitination-proteasome degradation by binding β-catenin, thereby enhancing cell resistance to Sorafenib [42]. In multiple myeloma, NEK2 inhibits its own and Beclin1 degradation by binding to ubiquitin-specific protease 7(USP7), thereby activating classical NF-κB signaling and autophagy pathways, promoting disease progression and resistance to bortezomib [41, 43]. Therefore, whether inhibiting *NEK2* affects Keap1 ubiquitination and proteasome degradation through ROS or other mechanisms needs to be further studied. NEK2 regulates Keap1 level through ubiquitination-proteasome degradation pathway, which then affects Nrf2 in cells and our study confirmed this phenomenon: inhibition of *NEK2* increased Nrf2 level, especially cell nucleus, and inhibition of Nrf2 decreased HMOX1 level. It is suggested that inhibiting *NEK2* to promote *HMOX1* transcription through Keap1/Nrf2 was an important mechanism to affect ferroptosis in gastric cancer cells.

In summary, this study found that *NEK2* could regulate *HMOX1* expression through Keap1/Nrf2, thus affecting the ferroptosis sensitivity of gastric cancer cells, enriching the pathological mechanism of gastric cancer and the role and mechanism of NEK2 in gastric cancer, providing evidence for the targeted inhibition of *NEK2* and then development of new therapeutic strategies for the prevention and treatment of gastric cancer.

## Supplementary Information

Below is the link to the electronic supplementary material.

Supplementary file1 (PDF 1169 KB)
**Figure S1.** AGS cell mycoplasma detection and STR identification. **A** AGS cell mycoplasma was tested negative; **B** The STR identification of AGS cells confirms no contamination.

Supplementary file2 (PNG 48 KB)

## Data Availability

All data generated or analyzed during this study are included in the article.
