# Supplementary material for: NEK2 affects the ferroptosis sensitivity of gastric cancer cells by regulating the expression of HMOX1 through Keap1/Nrf2
Source: Mol Cell Biochem. 2024 Mar 19;480(1):425–37. doi: 10.1007/s11010-024-04960-y (PMC11695390; doi:10.1007/s11010-024-04960-y)

AGS 细胞 STR 鉴定报告

检测方法：

用 Axygen 的基因组抽提试剂盒提取 DNA，采用 21- STR 扩增方案扩增，在 ABI 3730XL 型遗传分析仪上对 STR 位点和性别基因 Amelogenin 进行检测。

检测结果：

实验中阳性及阴性对照结果均正确，AGS 细胞的 STR 位点和 Amelogenin 位点的基因分型结果和图谱见附图。

分析说明：

AGS 细胞株基因组 DNA 扩增后，图谱清晰，分型结果良好。

鉴定结论：

该株细胞 DNA 分型在细胞系检索中找到**完全匹配**的细胞系，DSMZ 数据库显示细胞名为 **AGS**，细胞号对应 **CRL-1739**。本次检测在该细胞系中**没有发现多等位基因**。

附图 1：样本基因分型结果

细胞的 STR 位点和 Amelogenin 位点的基因分型结果						
Loci	送检细胞 STR 信息			细胞库细胞 STR 信息		
	送检细胞名：AGS			细胞库细胞名：AGS		
	Allele1	Allele2	Allele3	Allele1	Allele2	Allele3
D5S818	9	12		9	12	
D13S317	12	12		12	12	
D7S820	10	11		10	11	
D16S539	11	13		11	13	
VWA	16	17		16	17	
TH01	6	7		6	7	
AMEL	X	X		X	X	
TPOX	11	12		11	12	
CSF1PO	11	12		11	12	
D12S391	19	20				
FGA	23	24				
D2S1338	20	22				
D21S11	29	29				
D18S51	13	13				
D8S1179	13	13				
D3S1358	16	16				
D6S1043	12	12				
PENTAE	13	16				
D19S433	13.2	16				
PENTAD	9	10				
D1S1656	14	16				

附图 2：样本分型图谱

Applied Biosystems
GeneMapper ID v3.2

20221209

Sample File	Sample Name	Panel	OS	SQ
32_H04_Cellidentification-1-1212.fsa	AGS	21Plex_STR_Panel_v1.1		

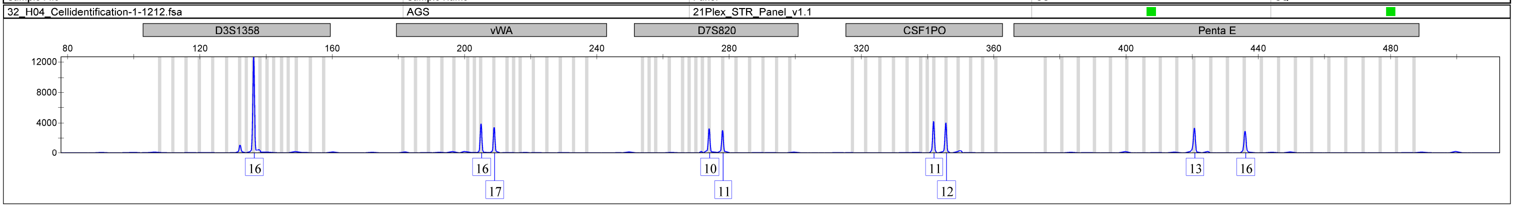

Sample File	Sample Name	Panel	OS	SQ
32_H04_Cellidentification-1-1212.fsa	AGS	21Plex_STR_Panel_v1.1		

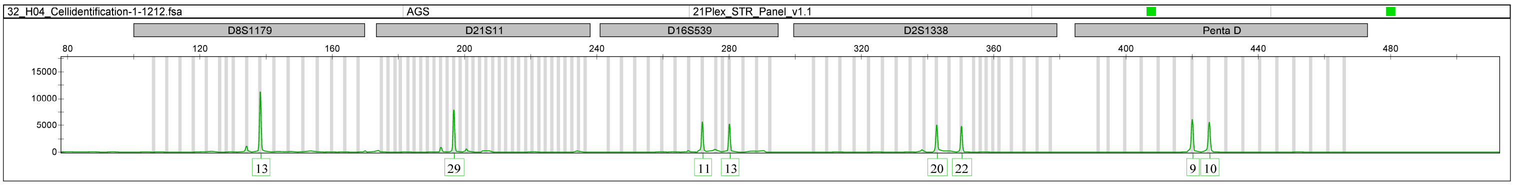

Sample File	Sample Name	Panel	OS	SQ
32_H04_Cellidentification-1-1212.fsa	AGS	21Plex_STR_Panel_v1.1		

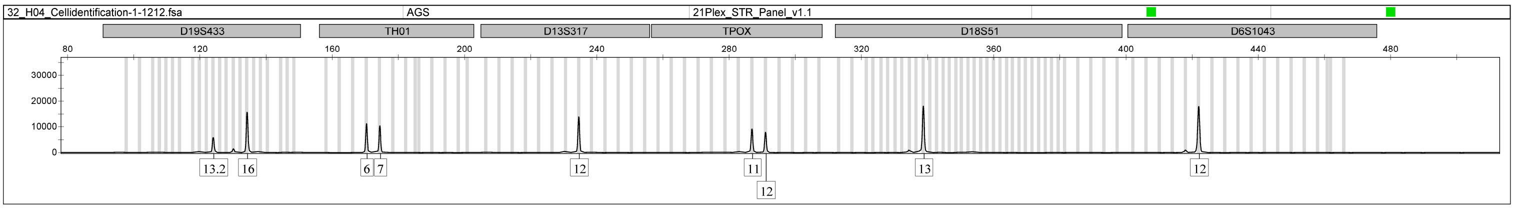

Sample File	Sample Name	Panel	OS	SQ
32_H04_Cellidentification-1-1212.fsa	AGS	21Plex_STR_Panel_v1.1		

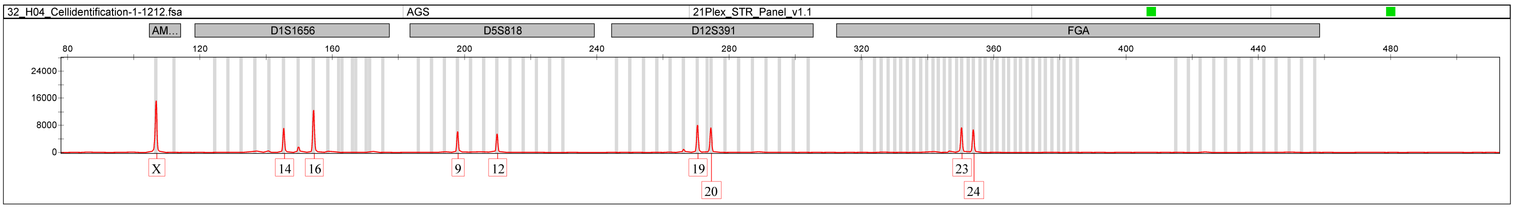

Supplement: Supplementary file 1 — Supplementary file1 (PDF 1169 KB) Figure S1. AGS cell mycoplasma detection and STR identification. A AGS cell mycoplasma was tested negative; B The STR identification of AGS cells confirms no contamination. [file 11010_2024_4960_MOESM1_ESM.pdf]
