# Supplementary figures and images for: NEK2 affects the ferroptosis sensitivity of gastric cancer cells by regulating the expression of HMOX1 through Keap1/Nrf2
Source: Mol Cell Biochem. 2024 Mar 19;480(1):425–37. doi: 10.1007/s11010-024-04960-y (PMC11695390; doi:10.1007/s11010-024-04960-y)

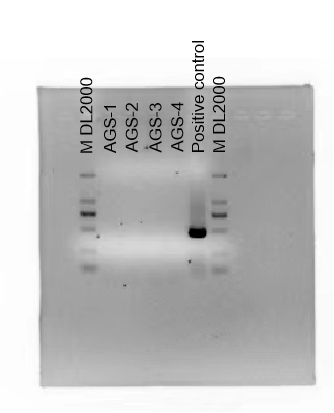

Supplement: Supplementary file 2 — Supplementary file2 (PNG 48 KB) [file 11010_2024_4960_MOESM2_ESM.png]
